# Integrated Analysis of Transcriptome and Metabolome in the Brain After Cold Stress of Red Tilapia During Overwintering

**DOI:** 10.3390/ijms252413372

**Published:** 2024-12-13

**Authors:** Chenxi Zhu, Haoran Yang, Wenbin Zhu, Qichen Jiang, Zaijie Dong, Lanmei Wang

**Affiliations:** 1 Freshwater Fisheries Research Centre of Chinese Academy of Fishery Sciences, Key Laboratory of Freshwater Fisheries and Germplasm Resources Utilization, Ministry of Agriculture and Rural Affairs, Wuxi 214081, China; zhuchenxi@hhu.edu.cn (C.Z.); y963021664@yeah.net (H.Y.); zhuwb@ffrc.cn (W.Z.); 2 School of Humanities, Universiti Sains Malaysia, Minden 11800, Penang, Malaysia; 3 Wuxi Fisheries College, Nanjing Agricultural University, Wuxi 214081, China; 4 Freshwater Fisheries Research Institute of Jiangsu Province, 79 Chating East Street, Nanjing 210017, China; qichenjiang@foxmail.com

**Keywords:** red tilapia (*Oreochromis* spp.), cold stress, brain, transcriptome, metabolome

## Abstract

Cold stress during overwintering is considered a bottleneck problem limiting the development of the red tilapia (*Oreochromis* spp.) industry, and the regulation mechanism is currently not well understood. In this study, the fish (initial weight: 72.71 ± 1.32 g) were divided into the cold stress group (cold) and the control (normal) group. In the control group, the water temperature was maintained at 20 °C, which is basically consistent with the overwintering water temperature in greenhouses of local areas. In the cold group, the water temperature decreased from 20 °C to 8 °C by 2 °C per day during the experiment. At the end of the experiment, the levels of fish serum urea nitrogen, glucose, norepinephrine, alkaline phosphatase, total bilirubin, and total cholesterol in the cold group changed significantly compared with that in the control group (*P* < 0.05). Then transcriptome sequencing and LC–MS metabolome of brain tissue were further employed to obtain the mRNA and metabolite datasets. We found that the FoxO signaling pathway and ABC transporters played an important role by transcriptome–metabolome association analysis. In the FoxO signaling pathway, the differentially expressed genes were related to cell cycle regulation, apoptosis and immune-regulation, and oxidative stress resistance and DNA repair. In the ABC transporters pathway, the ATP-binding cassette (ABC) subfamily *abca*, *abcb*, and *abcc* gene expression levels, and the deoxycytidine, L-lysine, L-glutamic acid, L-threonine, ornithine, and uridine metabolite contents changed. Our results suggested that the cold stress may promote apoptosis through regulation of the FoxO signaling pathway. The ABC transporters may respond to cold stress by regulating amino acid metabolism. The results provided a comprehensive understanding of fish cold stress during overwintering, which will facilitate the breeding of new cold-resistant varieties of red tilapia in the future.

## 1. Introduction

Tilapia is an African freshwater fish of the *Cichlidae* family, and it is an economically important aquatic animal for food security [1]. Red tilapia (*Oreochromis* spp.) is an excellent cross breed between the mutant red Mozambique tilapia (*O. mossambicus*) with other tilapia species such as Nile tilapia (*O. niloticus*) [2]. It has a huge market in many parts of the world due to its uniform red skin, the absence of black peritoneum, fast growth, and adaptability to any culture system [3]. However, cold stress during overwintering is the main problem limiting the development of red tilapia culture [3]. Red tilapia faces the problems of stunted growth, skin color darkening, and high mortality as a consequence of cold stress [4,5], so it is usually transferred into greenhouses during overwintering in some regions [6]. Red tilapia can tolerate low temperatures of 8 °C, with a critical temperature of 7 °C, and fish may die at 6 °C [7]. Although there have been many reports on the effect of temperature on tilapia, the exact regulatory mechanisms underlying the cold stress of red tilapia during overwintering are still unclear.

Temperature is an important stressor, especially in aquatic environments. However, many fish species, environments, regions, and aquaculture systems are vulnerable to extreme temperature (heat and cold) events caused by climate change. Aquatic ectotherms have difficulty adapting to large temperature fluctuations, especially in reproduction [8,9]. Cold stress will cause physiological and biochemical effects on fish. Acute cold exposure induced high oxidative stress in zebrafish liver, which may lead to mortality [10]. Cold stress could also lead to a decrease in blood cell count, DNA damage, and liver dysfunction, and increase the liver lipid metabolism, apoptosis, and inflammation-related gene expression in orange-spotted grouper (*Epinephelus coioides*) [11]. Exposure to acute cold stress conditions significantly enhanced plasma acid phosphatase activity and caused damage to the immune system of *O. mossambicus* [12]. Furthermore, the expression of proinflammatory cytokine genes was also regulated and immune properties were altered in the spleen, kidney, and liver of Nile tilapia chronically exposed to low temperatures [13]. Moreover, certain genes and metabolites are involved in the molecular responses to temperature stress [14,15,16].

Transcriptomic study is useful for the effects of low temperature on *Puntius tetrazona* [17], *Larimichthy crocea* [18], and *Pampus argenteus* [19]. In addition, metabolome has been used for the effects of low temperature on *Symphysodon aequifasciatus* [20] and *Nibea albiflora* [21]. However, there are limitations to using a single omics to study hypothermic stress in fish. Recently, the integrated analysis of transcriptome and metabolome in *E. sinensis* provided novel insights into the mechanisms of the precocity [22] and growth control during the molting cycle [23]. The comprehensive analysis of transcriptome, proteome, and metabolome of American shad (*Alosa sapidissima*) provided insights into the molecular regulation of high-temperature stress [16]. The multi-omics approach has provided a deeper understanding of the molecular mechanisms with diverse phenotypes. In this study, we attempted to explore the regulatory mechanism of cold stress in red tilapia by the integrated analysis of ranscriptome and untargeted liquid chromatography-mass spectrometry (LC–MS) metabolome. The findings of this study will provide a comprehensive understanding of fish cold stress during overwintering, which will facilitate the breeding of new cold-resistant varieties of red tilapia in the future.

## 2. Results

### 2.1. Fish Serum Physiological Parameters

At the end of the experiment, some red tilapia in the cold group ceased feeding when the temperature was below 10 °C and sank to the bottom of the tank and gradually lost equilibrium when the temperature was below 9 °C. But there was no mortality during the experimental period. For the serum indices, the levels of UREA (urea nitrogen), GLU (glucose), and NE (norepinephrine) of fish in the cold group were higher than those in the control group (*P* < 0.05) (Table 1). The levels of ALP (alkaline phosphatase), TBIL (total bilirubin), and TC (total cholesterol) of fish in the cold group were lower than those in the control group (*P* < 0.05) (Table 1). In order to further explore the specific regulatory mechanism of cold stress, we further conducted a combined transcriptome and metabolome analysis of brain tissue in red tilapia.

### 2.2. Differentially Expressed Genes (DEGs) Identification and Functional Enrichment

**Figure 1:**
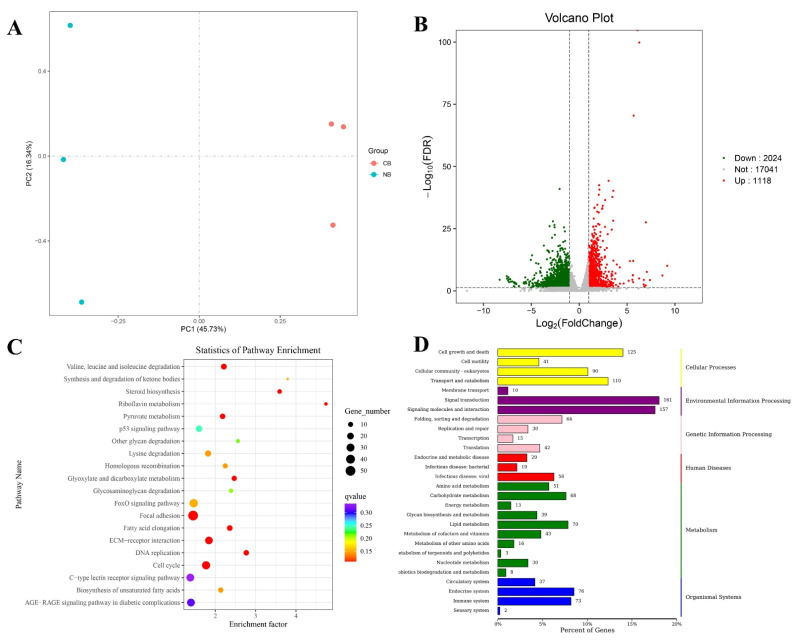
DEGs identification and functional enrichment. (**A**) PCA analysis, PC1 represents the first principal component, and the percentage in brackets represents the contribution rate of the first principal component to the sample difference; PC2 represents the second principal component; (**B**) volcano plot, each point in the volcano plot represents a gene. The *x*-axis represents the logarithm of the fold change in a gene expression in two samples, and the *y*-axis represents the negative logarithm of the FDR (false discovery rate). The greater the absolute value of the *x*-axis, the greater the fold change in the gene expression between the two samples. The larger the ordinate value, the more significant the differential expression, and the more reliable the differentially expressed genes (DEGs). The green dots represent down-regulated DEGs, the red dots represent up-regulated DEGs, and the black dots represent non-DEGs; (**C**) KEGG DEGs pathway enrichment scatter plot, the abscissa of the scatterplot is the enrichment factor, which represents the ratio of the number of target genes divided by the number of all the genes in each pathway. The larger the enrichment factor, the more significant the enrichment level of DEGs in this pathway. The color of the dot represents the q value, and the size of the dot represents the number of DEGs in the pathway; (**D**) KEGG DEGs classification map. The vertical coordinate (left) is the name of KEGG secondary metabolic pathway, the vertical coordinate (right) is the name of KEGG primary metabolic pathway, and the horizontal coordinate is the number of genes annotated to this pathway and their proportion to the total number of genes annotated.

In this study, six cDNA libraries of CB-4, CB-5, CB-6, NB-4, NB-5, and NB-6 in the cold stress (C) and control (normal) (N) groups were constructed and sequenced, with three bio-replicates of each sample. A total of 40.53 Gb data were obtained, with more than 6.44 Gb clean data of each sample. The Q30 ratio was higher than 92.3% for all samples (Supplementary Table S1). All the raw data were available in the NCBI SRA database with accession numbers SRR29147776, SRR29147782-SRR29147784, and SRR29147787-SRR29147788. All clean reads were compared with the reference genome, and the mapping rate was above 92.99%. All unigenes were compared with the databases for functional annotation, and 28,797 gene annotation results were obtained. PCA (principal components analysis) showed that the samples had good repeatability (Figure 1A).

With the FDR (false discovery rate) <0.05 and the fold change ≥1, we identified 3142 DEGs in the NB vs CB comparison group, which included 1118 upregulated genes and 2024 downregulated genes (Figure 1B). The detailed annotation and expression files of differentially expressed genes are available at Zenodo (13340318). As shown in Figure 1D of the KEGG annotation result, the biological processes of environmental information processing and cellular processes had the highest number of DEGs. In the environmental information processing, DEGs were most abundant in signal transduction, signaling molecules, and interaction. In the cellular processes, DEGs were most abundant in cell growth and death, transport, and catabolism (Figure 1D). As shown in Figure 1C of the KEGG enrichment result, the focal adhesion, FoxO signaling pathway, ECM-receptor interaction, and cell cycle were the main enrichment pathways.

### 2.3. Differential Metabolites (DMs) Identification and Functional Enrichment

**Figure 2:**
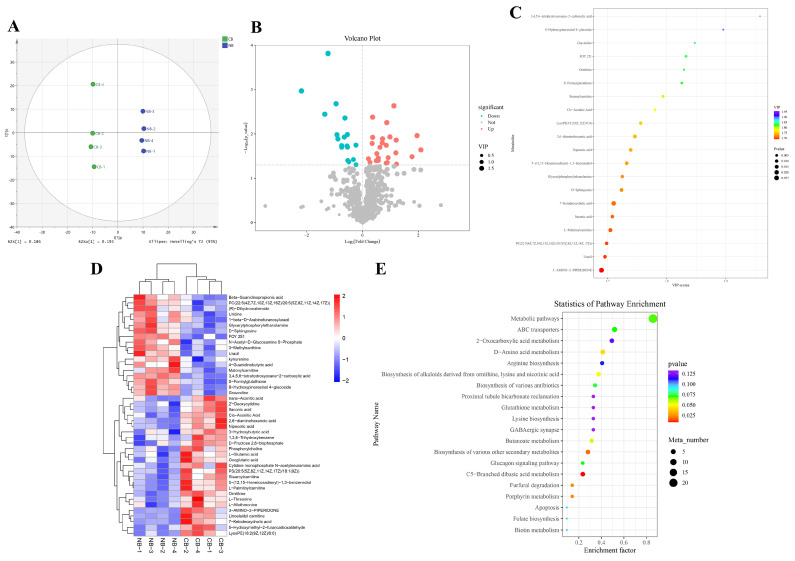
Differential metabolites (DMs) identification and functional enrichment, (**A**) orthogonal projections to latent structures-discriminant analysis score scatter plot of NB vs. CB. The horizontal coordinate t[1]p represents the predicted principal component score of the first principal component, and the vertical coordinate t[1]o represents the orthogonal principal component score, and the scatter shape and color represent different experimental groups. It can be seen that the difference between the two groups of samples is very significant, and all samples are within a 95% confidence interval (Hotelling’s T-squared ellipse). (**B**) Screening volcano plot of differential metabolites. Each point in the volcano plot represents a metabolite. The *x*-axis represents the fold change in the relative substances in the group (taking the logarithm of base 2), the *y*-axis represents the *p*-value of the Student’s *t* test (taking the logarithm of base 10), and the size of the scatter point represents the VIP value of the OPLS-DA model. The larger the scatter point, the larger the VIP value. Scatter colors represent the final screening results, with significantly upregulated metabolites shown in red, significantly downregulated metabolites shown in blue, and non-significantly differentiated metabolites shown in gray. (**C**) Top 20 VIP bubble map of differential metabolites. Each point in the bubble represents a metabolite. The *x*-axis represents the VIP scores of the top 20 differential metabolite variables, and the *x*-axis represents the top 20 differential metabolites. The scatter color represents the VIP value, and the scatter size represents the *p* value. (**D**) Differential metabolite clustering heatmap. The *x*-axis represents the sample name and the clustering result of the sample, and the *y*-axis represents the clustering result of the differential metabolites and substances. Different columns in the diagram represent different samples, and different rows represent different metabolites. The color represents the relative levels of metabolites in the sample. (**E**) KEGG DMs pathway enrichment scatter plot. Each row in the plot represents a KEGG pathway. The horizontal coordinate is the enrichment factor, and the larger the enrichment factor, the more significant the enrichment level of differential metabolites in this pathway. The color of the dots represents the *p* value, and the bubble size represents the number of differentiated metabolites annotated in the pathway.

In this study, the metabolome analysis of eight samples (CB-1, CB-2, CB-3, CB-4, NB-1, NB-2, NB-3, NB-4) was performed, with four bio-replicates of each sample in the NB vs CB comparative group. All the data in this study had been submitted to MetaboLights (MTBLS10298). After quality control and preprocessing (including filtering, missing value recoding, and normalization) of the raw data, multivariate statistical analysis including PCA and orthogonal projections to latent structures-discriminant analysis (OPLS-DA) was performed to screen the different variables. As shown in Figure 2A, the difference between the two groups of samples is very significant, and all samples are within a 95% confidence interval (Hotelling’s T-squared ellipse).

The study yielded totals of 43 (25 upregulated and 18 downregulated) DMs in the NB vs CB group (Figure 2B and Supplementary Table S2) with *P* < 0.05 and VIP (variable importance in the projection) >1. Then we selected the top 20 DMs for visualization in the form of a bubble map (Figure 2C), and found that the 3,4,5,6-tetrahydroxyoxane-2-carboxylic acid (downregulated), 8-Hydroxypinoresinol 4-glucoside (down), graveoline (down), ornithine (upregulated), S-Formylglutathione (down), FOY 251(down), and stearoylcarnitine (up) andcis-aconiticÂ acid (Up) deserve further attention and analysis. Figure 2D showed the heatmap of DMs. The KEGG enrichment analysis of the DMs in NB vs CB showed that the main enrichment pathways focused on the C5-branched dibasic acid metabolism, ABC transporters, porphyrin metabolism, furfural degradation, and D-Amino acid metabolism (Figure 2E). The details of the KEGG DMs annotation and pathway analysis results are shown in Supplementary Table S2.

### 2.4. DEGs Validation and Co-Analysis of DEGs and DMs

**Figure 3:**
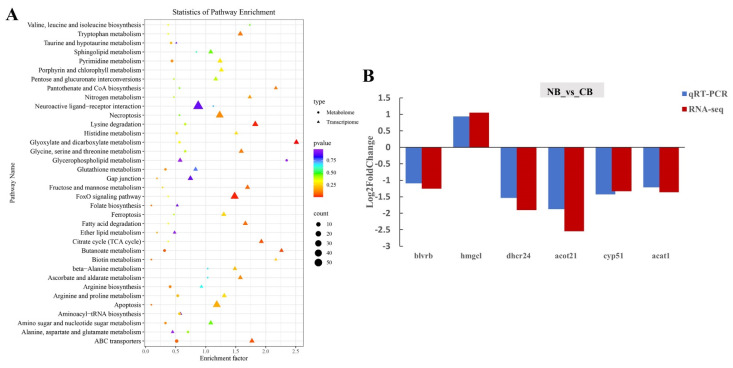
(**A**) KEGG pathway enrichment scatter plot of co-analysis of DEGs and DMs in NB vs. CB; (**B**) comparison of expression between qRT-PCR validation and Illumina sequencing. Log-fold changes are expressed as the ratio of gene expression after normalization to *β-actin*.

To validate the quality of the RNA-seq data, we randomly chose six DEGs, including biliverdin reductase b (*blvrb*), hydrophobically modified gelatin (*hmgel*), dehydrocholesterol reductase 24 (*dhcr24*), acyl-CoA thioesterase 21 (*acot21*), cytochrome P450, family 51 (*cyp51*), and acetyl-CoA acetyltransferase 1 (*acat1*) to validate the relative expression levels between the C and N group using real-time quantitative PCR (qRT-PCR). As shown in Figure 3B, the trends of the expression levels of these genes detected by qRT-PCR were the same as those obtained by RNA-seq data analysis. The results indicated the reliability of the RNA-seq data for the analysis of DEGs in this study. Gene primer sequence details are provided in Supplementary Table S1.

**Figure 4:**
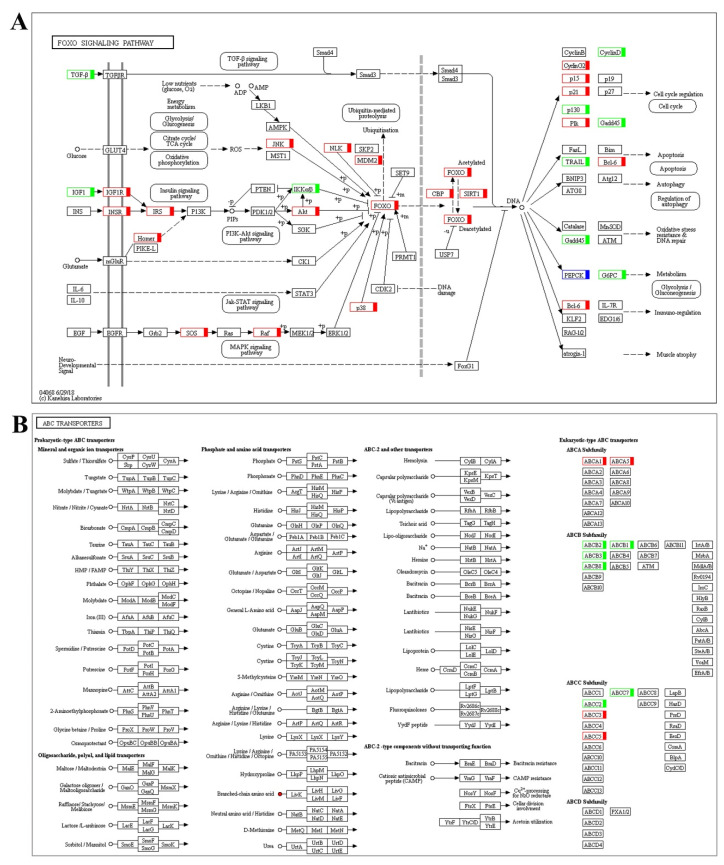
(**A**) FoxO signaling KEGG pathway; (**B**) ABC transporters KEGG pathway. Red indicates upregulation, green indicates downregulation, blue indicates both upregulation and downregulation.

We further performed the transcriptome and metabolome conjoint analysis. The results showed that the top 5 common pathways were the FoxO signaling pathway, glyoxylate and dicarboxylate metabolism, lysine degradation, apoptosis, and ABC transporters in NB vs. CB (Figure 3A). In the FoxO signaling pathway, 34 gene expression levels and 1 metabolite (L-Glutamic acid) were significantly changed. And the upregulated genes, including c-Jun N-terminal kinase (*jnk*), protein kinase B (*akt*), *p38*, *p15*, *p21*, *cyclin G2*, and Polo-like kinase (*plk*), were all related to cell cycle regulation; the b-cell lymphoma 6 (*bcl-6*) gene was related to apoptosis and immune regulation. The glycolysis-related gene phosphoenolpyruvate carboxykinase 1 (*pck1*) was upregulated, but the *pck2* gene was downregulated. The oxidative stress resistance and DNA repair related gene growth arrest and DNA-damage-inducible 45 (*gadd45*) was downregulated (Figure 4A). In the glyoxylate and dicarboxylate metabolism pathway, 11 genes were up or downregulated and 2 metabolites were significantly changed. The oxoglutaric acid metabolite was upregulated, but the DEGs were almost all downregulated. In the ABC transporters pathway, the ATP-binding cassette (ABC) subfamily ABCA, ABCB, and ABCC gene expression levels, and the deoxycytidine, L-lysine, L-glutamic acid, L-threonine, ornithine, and uridine metabolite contents changed (Figure 4B).

## 3. Discussion

A decrease in water temperature exposes fish to cold stress, which can adversely impact their physiology, behavior, and survival [24]. Under experimental conditions, the change in blood indices can provide valuable information about the physiology and health status of fish [25]. In this study, cold stress led to a significant decrease in ALP, TC, and TBIL levels, while an increase in GLU, NE, and UREA levels in the serum of red tilapia ALP plays an important role in immune and metabolic pathways in fish [26]. Studies indicated that the decrease in ALP activities and TBIL was favorable for liver protection [27,28]. TC is an indicator of lipid metabolism. Blood GLU is commonly used as a stress indicator in fish, and the increase in the serum GLU content may be a demand for energy provision against cold stress in this study [11]. We also found that the content of UREA increases under cold stress; the same results of sea bream (*Sparus aurata*) liver [29] and yellow catfish (*Pelteobagrus fulvidraco*) serum [30] indicated that purine metabolism may be induced, and the levels of uric acid and hypoxanthine were upregulated under cold environments. NE acts as a regulator of homeostasis in response to environmental stress. Chang et al. [31] demonstrated that NE stimulated immunocompetence parameters and related gene expression during acute stress and exerted a regulatory effect on SOD activity. It can be seen that cold stress may affect the immune antioxidant function and metabolic capacity in red tilapia.

By combined transcriptome and metabolome analysis, we found that the FoxO signaling pathway, glyoxylate and dicarboxylate metabolism, lysine degradation, apoptosis and ABC transporters pathways played an important role in the cold stress. FoxO signaling regulates cell survival, apoptosis, and DNA repair [32]. Thus, it plays an important role in the regulation of tissue homeostasis under stress conditions [33]. In this study, cold stress led to significant changes of 34 gene expression levels and 1 metabolite in the FoxO signaling pathway. And the upregulated genes, including *jnk*, *akt*, *p38*, *p15*, *p21*, *cyclin G2*, and *plk* were related to cell cycle regulation; the *bcl-6* gene was related to apoptosis and immune regulation. Apoptosis plays a crucial role in maintaining cell homeostasis, immune defense, and development in fish. Oxidative damage caused by low-temperature stress can elevate apoptosis by influencing the expression of apoptosis-related genes [34]. In tilapia, fish fell into coma when apoptosis signals began to appear, and tilapia died when about 75% cells underwent apoptosis [35]. Furthermore, biological pathways, including FoxO signaling and metabolic regulation, were suggested to account for the differential low-temperature limit between Nile tilapia and zebrafish (*Danio rerio*) [35].

In this study, the glycolysis-related gene *pck1* and glutamic acid metabolite were up-regulated, which may be in favor of glucose production. Correspondingly, the level of fish serum GLU in the cold group was higher than that in the control group. The liver plays a critical role in maintaining systemic glucose homeostasis by regulating glucose uptake, utilization, gluconeogenesis, and glycogenolysis. These processes are primarily controlled by key enzymes, including glucokinase (GCK), PCK1, and glucose-6-phosphatase (G6PC), which are essential for hepatic gluconeogenesis [36]. ABC transporters, including oligosaccharide, polymer, lipid, monosaccharide, phosphate, and amino acid transporters, catalyze ATP hydrolysis to drive molecular transport using the energy released [37]. In this study, it was found that the metabolic pathway, C5-branched dibasic acid metabolism, microbial metabolism in diverse environments, and ABC transporters pathways were significantly enriched in NB vs CB, which may have a protective effect on fish cold stress adaptation. And the metabolites of L-lysine, L-glutamic acid, L-threonine, and ornithine contents in the ABC transporters pathway changed in the fish of the cold group. The reason for this phenomenon may be that cold stress hindered the metabolism of amino acids, or increased levels of phosphate and amino acids resisted cold stress. ABC transporters were also induced under cold stress in the liver of *Nibea albiflora* [21]. Recent research has found that black porgy (*Acanthopagrus schlegelii*) enriched in glycine, serine, and threonine metabolism, aspartate and valine metabolism pathways in response to low temperature stress, and the downstream amino acids metabolism was also downregulated [38]. Further studies need to be conducted to make more discoveries.

## 4. Materials and Methods

### 4.1. Ethics Statement

All animal experiments in this study were approved by the Bioethical Committee of Freshwater Fisheries Research Center (FFRC) of the Chinese Academy of Fishery Sciences (CAFS) (BC 2013863, 9/2013), guidelines for the Care and Use of Experimental Animals of China.

### 4.2. Fish and Experiment

The red tilapia used in the study was a new aquaculture variety “Zhongheng No. 1” (GS-01-002-2022) with genetically stable and consistent pink skin color. The fish were kept in indoor conical fiberglass tanks (diameter 150 cm × depth 120 cm) and acclimated in a flow-through water system for overwintering. A total of 80 fish (initial weight 72.71 ± 1.32 g) were randomly divided into C and N groups (40 fish in each group). During the experiment, the room temperature was less than 5 °C. So, the water temperature (WT) was maintained at 20 °C by a temperature controller (SUNSUN, Zhejiang, China) in the control (normal N) group, which is basically consistent to the overwintering water temperature in greenhouses in local areas. In the C group, the WT was decreased by 2 °C per day from 20 °C to 8 °C. Every morning at 8 a.m., the temperature controller was set 2 °C lower. During the experiment, the same feed (Tong Wei, Chengdu, China) was used, and the fish were fed twice a day until the day before sampling. Aeration was supplied to each tank 24 h per day, and the photoperiod was 12 D:12 L. During the experimental period, the swimming and feeding behaviors of fish were observed and recorded every day.

The period of the experiment was 7 days. On the 7th day, 6 fish were randomly selected and sampled from the C and N groups, respectively. The fish were initially anesthetized using a 20–30 mg/L MS-222 buffer, and the brain tissue of fish were excised quickly and snap-frozen in liquid nitrogen and stored at −80 °C until processed. Blood was taken from the caudal vein of fish with syringes, and serum samples were obtained after centrifugation (3000× *g* for 15 min) at 4 °C for ALP, aspartate transaminase (AST), albumin (ALB), creatinine (CREA), cortisol, GLU, total protein (TP), TBIL, UREA, TC, triglyceride (TG), epinephrine (EPI), NE, catecholamine (CA), cortisol, and thyroxine (T4).

### 4.3. Transcriptome and Metabolome Analysis

The main analysis process of transcriptome sequencing involves RNA extraction, library construction and sequencing, library quality control, and bioinformatics analysis (including functional annotation, DEGs identification, and enrichment analysis).

The RNA of brain tissue was extracted using the TRIZOL (Invitrogen, Carlsbad, CA, USA) method. Next, 6 cDNA libraries of CB-4, CB-5, CB-6, CB-4, CB-5, and CB-6 were constructed. The library preparations were sequenced on an Illumina Nova seq6000 platform for 150 bp paired-end sequencing. The quality control of raw data (raw reads) was performed with Fastp software (v0.20.0). The clean reads were aligned to the reference genome using HISAT2 (v2.1.0). Reference genome version: *O_niloticus*_UMD_NMBU. Reference genome website: http://ftp.ensembl.org/pub/current_fasta/oreochromis_niloticus/dna/ (accessed on 15 August 2024). Then StringTie was used to assemble transcripts. StringTie was used again to quantify gene expression according to the annotation file. The PCA analysis was performed using the R (V3.6.2) prin_comp_data function. Differential expression analysis of the cold stress and control groups was performed using the DESeq2 (v1.26.0) R package. Genes with an adjusted *p*-value < 0.05 were assigned as differentially expressed. FDR <0.05 and fold change ≥1 was set as the threshold for significantly differential expression in this study. The detailed information is shown in [22,39].

Differential metabolites in samples of the cold stress and control groups (including four independent biological replicates) were detected by untargeted metabolomic analysis. The detailed methods are described in [22]. The main processes included metabolite extraction, LC–MS analysis, data preprocessing, and annotation. LC–MS/MS analysis was conducted using a Q Exactive Orbitrap UHPLC system (Thermo Fisher Scientific, Waltham, MA, USA). DMs were identified based on a VIP score greater than 1 and a *p*-value less than 0.05. Enriched KEGG pathways were analyzed using KOBAS and Fisher’s exact tests.

### 4.4. qRT-PCR Validation of DEGs

RNA samples from the brain tissue of three fish used for the RNA-seq were analyzed by qRT-PCR. As described by [39,40], qRT-PCR was performed on a CFX96 Real-Time PCR System (Bio-Rad, Hercules, CA, USA). The final volume of each PCR reaction was 25 μL. The relative mRNA expression levels were normalized to *β-actin* and calculated using the 2^−ΔΔCt^ method.

### 4.5. Conjoint Analysis of Transcriptome and Metabolome Data

As shown in [40], the Spearman correlation coefficient was used to do the integrative analysis of transcriptome and metabolome data of the cold stress and control groups in this study. We first screened the top 200 DEGs in the transcriptome. The Psych package in R was used to calculate the Spearman correlation between the top 200 DEGs and the top 50 differential metabolites. Correlations with a value greater than 0.9 and a *p*-value less than 0.01 were further filtered and used to construct the maps.

### 4.6. Statistical Analysis

Statistical analysis was performed using SPSS 25 (SPSS Inc., Chicago, IL, USA). The data conformed to normal distribution and variance chi-square tests. Significance of differences were analyzed using the independent samples *t*-test. All the results are presented as mean ± standard error of mean (SEM).

## 5. Conclusions

Cold stress during overwintering is considered a bottleneck problem limiting the development of the red tilapia industry, and the regulation mechanism of cold stress is currently not well understood. In this study, cold stress changed some serum physiological parameters of fish. Then, transcriptome sequencing and LC–MS metabolome of brain tissue were further employed to explore the specific regulatory mechanism. We found that the FoxO signaling pathway and ABC transporters played an important role. The cold stress may promote apoptosis through regulation of the FoxO signaling pathway. The ABC transporters may respond to cold stress by regulating amino acid metabolism. Corresponding DEGs and DMs were identified. Our results provide a comprehensive understanding of fish cold stress during overwintering, which will facilitate the breeding of new cold-resistant varieties of red tilapia in the future.

## Figures and Tables

**Table 1:** The serum physiological parameters of red tilapia in the cold and control groups. Values represent means ± SEM (n = 3).

Items	Cold	Control	Significance
AST ^1^	648.43 ± 18.88	554.13 ± 53.69	
ALP	14.60 ± 0.12	27.45 ± 0.66	**
T-BIL	6.63 ± 0.63	11.83 ± 0.64	**
TP	34.64 ± 1.94	32.56 ± 0.54	
ALB	14.02 ± 0.88	13.36 ± 0.23	
UREA	0.96 ± 0.08	0.59 ± 0.08	*
TC	4.54 ± 0.05	5.34 ± 0.30	*
TG	1.95 ± 0.09	1.68 ± 0.16	
CREA	13.93 ± 1.38	16.93 ± 0.82	
GLU	10.47 ± 0.22	8.23 ± 0.41	**
T4	13.07 ± 0.38	13.27 ± 0.61	
Cortisol	94.75 ± 1.86	94.45 ± 2.95	
EPI	9.47 ± 0.52	8.61 ± 0.26	
NE	74.33 ± 1.15	64.95 ± 4.10	*
CA	98.65 ± 5.35	92.08 ± 5.57	

^1^ AST: aspartate transaminase; ALP: alkaline phosphatase; T-BIL: total bilirubin; TP: total protein; ALB: albumin; UREA: urea nitrogen; TC: total cholesterol; TG: triglyceride; LDH: lactate dehydrogenase; CREA: creatinine; GLU: glucose; T4: thyroxine; EPI: epinephrine; NE: norepinephrine; CA: catecholamine. * Indicates a significant difference between groups (*P* < 0.05). ** Indicates a significant difference between groups (*P* < 0.01).

## Data Availability

The original contributions presented in the study are included in the article/Supplementary Material, further inquiries can be directed to the corresponding authors.

## Supplementary Materials

The following supporting information can be downloaded at: https://www.mdpi.com/article/10.3390/ijms252413372/s1.
