# Supplementary material for: Integrated Analysis of Transcriptome and Metabolome in the Brain After Cold Stress of Red Tilapia During Overwintering
Source: Int J Mol Sci. 2024 Dec 13;25(24):13372. doi: 10.3390/ijms252413372 (PMC11676689; doi:10.3390/ijms252413372)
Supplement: Supplementary file 1 [file ijms-25-13372-s001.zip › Table S1 Basic informations of the transcriptome and gene primer sequence details.pdf]

Basic informations of the transcriptome.

Sample	ReadSum	BaseSum	GC(%)	N(%)	Q20(%)	CycleQ20(%)	Q30(%)
CB-4	21968833	6590649900	44.68	0	97.31	100	92.71
CB-5	22171972	6651591600	44.41	0	97.42	100	92.88
CB-6	25239118	7571735400	45.8	0	97.14	100	92.3
NB-4	21912154	6573646200	44.42	0	97.23	100	92.5
NB-5	22334504	6700351200	44.37	0	97.16	100	92.33
NB-6	21466915	6440074500	44.42	0	97.5	100	93.05

Gene primer sequence details.

Genes	Forward primer	Reverse primer	Product length
blvrb	ACAAGGCATCCAGAGTGGTG	GTAGTTGGGCTGAGGTCGTT	121
hmgcl	CAAGTCCTCACCCCCAATCTC	TAACCTCTAACTGGCACACCG	191
cyp51	CAAACAGTCCACAGACGAGGA	TCCAGAAACTCAATGGGGCTC	108
dhcr24	TCTTGGTTGCCGCTGAGATT	AATGTGTTCCGCTTGTTCGC	130
acat1	TCTGTGCCTCTGGAATGAAGTC	TGAGTCCGTCCTTCACAATGAG	180
acot21	GGAAGCGACCAGAGGTGAAA	GCAGTGGCGGGAATACAATG	180
